# Indents

**Published:** 1921-07

**Authors:** 


### INDENTS

**☞Brief Articles of Technical or Scientific Value will receive notice and comment. Contributions invited.**

---

SORE MOUTHS FROM VULCANITE PLATES. I have come to the conclusion that a great deal of the so-called rubber sore mouth and the softening of the alveolar process under the rubber plate is due to the lack of cleanliness. Some patients who are most fastidious about their personal cleanliness give very little attention to their mouths, in fact never properly clean their plates.

I go to the trouble of buying my patients a special brush and presenting it to them. I advise them to get a piece of ordinary laundry borax soap, something that will cut the grease and then finally to polish the plate with bicarbonate of soda. I know it is lack of cleanliness that causes the softening of teeth under clasps. If patients will take the end of a match stick and polish the inside of their clasps with bicarbonate of soda, they will not have the enamel wear. I have seen clasps worn for fifteen years without the slightest sign of abrasion, and in every instance the patient had kept the clasps clean with bicarbonate of soda or some tooth-paste. No doubt in the future we shall hear a great deal about loss of teeth on account of clasps, but it will be due mostly to the fact that the patient did not receive the proper instruction as to the cleaning of them, and again to the fact that the dentist does not observe the fundamental principles of clasp construction. Most of the modern cast clasps are so rough on the surface next to the tooth that it is almost impossible to keep them clean.

I think we have a great responsibility resting on our shoulders in this matter of mouth hygiene. We must give more time to instructing our patients as to the proper care of the mouth.—Dr. Kennedy, in *Cosmos*, June, 1921, Page 641.

---

ORAL HYGIENE SCHOOL PHYSIOLOGIES. Oral hygiene has placed orders for physiologies with every first-class school book publisher in the United States.

As rapidly as possible these books will be searched for errors.

The object of this campaign is to show the need of expert dental advice in the preparation of the chapters upon the mouth and its contents.

The State of Missouri has already led the way by passing a law which requires the school physiologies that are used in the public schools of that state to be approved by a committee from the State Dental Association and the State Board of Health.

In each state, those who practice the healing arts must show their qualifications before a board of competent examiners. This is done to protect the public and to stimulate a higher degree of professional education.

Is it not reasonable to have a committee, from those who are qualified to practice dentistry, pass upon the teachings of mouth hygiene and anatomy that are found in the public school books?

This can be done in two ways—first, by laws in the various states, or even by rulings of the boards of education, and second, by a committee from the National Dental Association that will have authority to endorse any school physiology in which the text agrees with the scientific facts that are known to modern dentistry.

The school book publishers are vitally interested in education. The reason they have never submitted the texts upon this subject to such a committee is because there has never been an authoritative body to consult.

Both economy and efficiency would be greatly enhanced by their being able to print the endorsement of the National Association in their books. Other endorsements are so used, why not ours?

Such a committee should be composed of able men who are neither narrow nor bigoted—real representatives of the intelligence of our Association.

If the National Dental Association will take this matter up in the right spirit they will be met more than half way by the publishers, and the state dental associations can very quickly bring the state boards of education to a realization of the propriety of making adoptions that will coincide with the practical work that is being done in the public school clinics.

Before this series of editorials ends, a list of the medium-good and the excellent school physiologies will be printed.

Energy and books are too expensive to be wasted. Let us do our part to see that the energy that is put into lessons is not wasted and to see that the mouth, as a factor in health, is well presented in the school physiologies.

Here is a chance to start a real educational propaganda that all can join.—*Editor Register.*

---

Conservative Dentistry. If all the human beings, who are living at the present time with pulpless teeth in their jaws, would have to expect in the future to be as sick as some members of the medical and dental profession are trying to make us believe they will be in due time, I am afraid the human race will near its end very soon.

I am just as conservative, although financially it may be to the disadvantage of an oral surgeon; but I believe we must be conservative and have faith in the good dental work which is done by the profession.

If a person comes to my office or to the clinic who is in good health, I personally feel that the pulpless tooth may be retained and treated by dental means. On the other hand, if the patient is troubled with some systemic trouble, and physicians have made a thorough examination and have not found a cause anywhere for this systemic disorder, I feel if pulpless teeth are found they should be either extracted, or if possible, subjected to dental treatment.

The radical views which are taken by some men in our profession, because a number of cases have gotten well after the removal of pulpless teeth, I feel are not logical. The teeth as such are just as important organs in the human body as any other, and therefore an attempt should be made to save them if possible.

Dr. Carl Lucas made a statement to me that in curetting apical areas he not only removes the granulation tissue, but also the bony wall, and curets the cancellous tissue of the bone beyond it, making the statement that he gets positive cultures from the areas beyond the actual rarefaction. While this is quite possible, I believe that it does not warrant the breaking of the bone wall which nature has put around an apical area. It is only natural that during an operation, even if one is very careful, some of the bacteria present will be carried beyond the bony wall, and therefore the infection of the cancellous tissue is the natural result. If bacteria were not able to travel through the granulation area the danger of focal infection of course would not be present at all.

I do not think anyone can make the statement that with a perfectly filled root canal such an area can not be reinfected. I know that a great many oral surgeons besides myself have had to remove teeth which apparently, according to the X-ray, were perfectly filled. I quite agree that a radiographer, whether medical or dental or what not, is not in a position to give advice as to treatment. My belief is that a radiographer should do nothing else but read what the X-ray shows. That means he should tell the practitioner his findings according to the

X-ray; but he is in no position to give advice as to treatment, without actually examining the case and knowing the history.

We are unfortunately burdened in New York with just that type of radiographers—one in particular, a medical radiographer, who has a wonderful chart with thirty-two diagnoses and outlines of treatments. On this chart under No. 30, for instance, you will find "Roots should be amputated." Under another number, he says the "Tooth should be removed." It is to my mind arrogance on the part of the radiographer to tell the practitioner what treatment to instal in a certain case.—Theodore Blum.

---

Correcting Artificial Teeth. To reproduce a piece of work that will be in perfect harmony with the neighboring teeth, that the type must be held in mind throughout the individual case.

Unfortunately, manufacturers of facings or dowel crowns have not a sufficient number of molds to take care of the different types of teeth, and it becomes necessary for the operator to change the contour of the teeth if an artistic piece of work is to be produced.

I shall endeavor to explain in a brief way how this can be accomplished. Let us bear in mind that the average tooth fuses at approximately 2460° F. We can not use high-fusing 2560° porcelain without destroying the tooth itself. We must use a porcelain that has a lower fusing point than that of the porcelain tooth. For our purpose we use an outfit of the S. S. White crown and bridge porcelains, which comes in a box containing twenty-five colors; the fusing point of these porcelains is 2300° F. The shade guide which accompanies the outfit includes enough to enable us to match almost any color, and in very few instances will it be necessary to combine colors of the shade guide to perfectly match a tooth.

If it is necessary to add a contact point, or to change a facing, or to build gingival corrections to a tooth, the porcelain is mixed and manipulated in the same manner as for jacket crowns and inlays.

It is a good policy to first study the type of tooth we are going to reproduce, fix that firmly in our minds and then duplicate just that type. A color may be a degree off shade and the work still be correct if the type harmony of the rest of the teeth has been reproduced. When the tooth has been properly built up, place it in a cold oven, gradually carry the heat to the fusing point, 2300°, then turn the current off, and allow the tooth to cool within the oven. If it is necessary to bake the crowns more than once, the fusing point of the porcelain must not be reached, but a heat of about 100° below should be the stopping point until the case is ready for the final fuse.

There is the exception, of course, in using an Ash tube tooth. The Ash tooth is fused at about 2250°, therefore a lower fusing porcelain, such as Brewster's or Jenkins', should be used to change the contour.

Very few artificial teeth have perfect occlusion. This is especially true of premolars and molars. Cusps should always be added to these teeth to obtain perfect articulation with the opposing teeth. This can always be accomplished by the use of the lower fusing porcelain.—F. R. Felcher, in *Cosmos*, June, 1921, Page 631.

---

Novocaine a Skin Irritant. My experience with novocaine, published in the December, 1920, Dental Cosmos, under the title "Novocain as a Skin Irritant," called forth some very interesting letters from different parts of the country.

Some dentists had already found that novocain was the cause of dermatitis, others had not. One had sold his practice and gone to the Mayo Clinic for treatment, where it is recognized that novocain is a cause of dermatitis among dentists.

I recently attended a lecture at the Forsyth Infirmary by Dr. Guy Lane, Boston, who has done some investigating on novocain as a cause of dermatitis among dentists.

Most dentists seem skeptical and not inclined to believe that such a condition can be brought about by the use of novocain. The extreme degree of my own affection was caused by not knowing that it is a skin irritant to me, and continuing its use over a period of months. Had I known that it is a possible cause of dermatitis, I could have protected myself earlier, and saved a whole season of misery.

As I am one of the earlier ones to have been affected by it, having given the subject considerable study, and having during the past year used more novocain than ever before without any trouble, I thought possibly my further experience might be of interest.

I do not think that novocain affects the great majority in this way, only an unfortunate few who are susceptible to it. I do not think any one, whether susceptible or not, need be afraid of it or discontinue its use. If one's hands or face become irritated consider the possibility of novocain as a causative factor, giving due consideration to other possible causes, as Howe's formalin-silver solution, X-ray developing solutions, etc. If it is proven that novocain is the cause, as in my own case, adopt a good glove technic. I have reports from a few who after using gloves were not entirely free from irritation. At first I had this trouble, but when irritation appeared I made a study to find the break in my technic, and never failed to find that I had broken the line somewhere. It is absolutely necessary that the hands shall not, at any time, come in contact with the solution.

I use a loose glove that slips on easily to economize time. After making the injection I thoroughly wash the gloves in soap and water, dry and powder, and remove by turning inside out and using left for right next time. As the gloves are worn only for protection the tendency is to get careless about thoroughly washing before removing from the hands. If any attempt is made to remove the gloves before washing, the hands are liable to come in contact with the contaminated surface of the gloves. Do not touch the syringe, dissolving cup, or any thing that has been in contact with the solution, before putting on the gloves. Do not forcibly expel the air bubble from the syringe because it fills the air with a fine spray that will come in contact with the face. It must be a perfect unbroken technic at all times.

After injection unless I am working on infiltrated tissue I have the patient rinse the mouth, and work with bare hands to either operate or extract, without any skin irritation.

I had novocain injected for an extraction with perfect results, and no skin irritation following the injection.

It is a source of much satisfaction to me to have established beyond the possibility of doubt the cause of my trouble and that it can be so easily controlled.—Dr. E. Guptill, in *Cosmos*, June.

---

ETHICAL PARABLES. At last the philosopher found the dentist who could answer his question as to what constituted a demonstration of ethics in dental practice. He was quiet, easy mannered and apparently prosperous.

"The application of ethics to practice," said he, "includes far more than we were taught in college. It consists in knowing what service costs, and selling it at fees that make it possible for the dentist to meet all his obligations by a number of working hours which will not affect his health; which will place his own financial affairs in such shape that they will not cause him anxiety nor thrust themselves into his working time; which will allow him to attend the necessary meetings and possess the proper books and magazines; and enables him to give to each patient his undivided and unhurried attention as the case requires.

"The dentist might do all these things and still be unethical by withholding from patients something that was their due.

"Ethics is part of the spirit of a man. If the man is over-worked, hurried in his service, worried by unfavorable financial conditions, and hopeless of material achievement for himself, ethics will get small chance for expression in service, however great his desire.

"If the man will first organize his own affairs to permit rest, leisure, aspiration and deliberate service, he may be ethical if he will.

"Ethics is the expression of a fine spirit, working unhindered, in helpful service."—George Wood Clapp, in *Digest.*

---

Quick Repair for Porous Places in Plates. Whenever you desire a quick repair for porous place in plate, cut out that part, save the fine powder, mix powder with synthetic porcelain, then fill cavity. After this has solidified and is polished it is very difficult to detect place repaired. Use rubber filings from some plate you are to repair.—C. I. Faison, in *Digest.*

